# Comparative Study on Knowledge About Autism Spectrum Disorder Among Paediatric and Psychiatric Nurses in Public Hospitals in Kumasi, Ghana

**DOI:** 10.2174/1745017901814010099

**Published:** 2018-03-30

**Authors:** Wireko-Gyebi Sampson, Ashiagbor Emelia Sandra

**Affiliations:** 1 University of Energy and Natural Resources, Sunyani, Ghana, West Africa; 2 Ashanti School for the Deaf, Jamasi, Ghana, West Africa

**Keywords:** Autism spectrum disorder, Knowledge of ASD, Paediatric nurses, Psychiatric nurses, Public hospitals, Kumasi

## Abstract

**Background:**

Despite the existence of autism spectrum disorder in Ghana, few studies have provided the necessary information on the phenomenon. These studies have mostly focused on speech and language therapy for children and modification of classroom environment for children with autism spectrum disorder. This approach has resulted in a paucity of knowledge on nurse’s knowledge of autism spectrum disorder in Ghana.

**Objective:**

The study sought to assess the knowledge of paediatric and psychiatric on autism spectrum disorder.

**Method:**

In this study, 130 paediatric and 93 psychiatric nurses sampled from five public hospitals in the Kumasi Metropolis participated in the survey. The Knowledge about Childhood Autism among Health Workers (KCAHW) questionnaire was employed to assess their knowledge of autism spectrum disorder.

**Results:**

It emerged from the study that psychiatric nurses were more knowledgeable on autism spectrum disorder than paediatric nurses in general and specifically on each of the four domains on the KCAHW questionnaire. However, the level of knowledge on autism spectrum disorder among both groups of nurses remains low. Apart from the previous encounter, there were no significant differences between paediatric and psychiatric nurses’ gender, age, marital status, working experience and their knowledge.

**Conclusion:**

In view of the findings, it will be appropriate for autism spectrum disorder to be included in the clinical training curriculum as well as the continuous professional education for both paediatric and psychiatric nurses. This will go a long way in helping paediatric and psychiatric nurses to increase their knowledge of autism spectrum disorder.

## 1 INTRODUCTION

The American Psychiatric Association (APA) defines Autism Spectrum Disorder (ASD) as a spectrum of neurodevelopmental disorders occurring early in childhood that is characterized by persistent deficits in social communication and interaction and restricted, repetitive patterns of behaviour, interests, or activities [1]. Studies have shown that children with ASD exhibit three main developmental deficits: altered social interaction, inability to communicate verbally and nonverbally and repetitive behaviours such as obsessive interests [2]. Additionally, Inglese MD, Elder JH. as well as Schnur J. have cited sensory problems, sleep disturbances, poor communication and interaction, social isolation and trouble with new situations adaptation as the most common and prevalent characteristics of children suffering from the disorder [3, 4]. Dominick KC *et al*. posited that children with ASD do not only have language problems but also exhibit tantrums, self-destructive acts and other forms of inappropriate public behaviour [5].

According to Ballentine F., ASD is common neurodevelopmental disorders that are gaining attention [6]. This can be attributed in parts, to the fact that the prevalence of the disease is on the ascendancy. A worldwide epidemiological survey conducted indicates that the prevalence of ASD across the globe stands at 62 per 10,000 children [7]. This figure increased astronomically in 2013. The World Health Organisation [8] indicated that ‘epidemiological data estimate the global prevalence of ASD to be one person in 160, accounting for more than 7.6 million disability-adjusted life years and 0.3% of the global burden of disease’. Statistics at the Centre for Disease Control and Prevention (CDC) postulate that ASD is prevalent in the United States. Accordingly, Inglese MD, Elder, JH. opined that majority of the United States population either has a family member or knows of someone diagnosed with ASD. Centers for Disease Control and Prevention indicated that 1 in every 110 children in the United States is affected by the disorder [9]. Currently, the prevalence rate is about three to four boys to one girl. Sanua VD opined that ASD is largely a disorder of children living in the Western World and may not be common in Africa [10]. However, earlier studies [11, 12] revealed the presence of the disease in some African countries such as Ghana, Nigeria, Kenya, Zimbabwe, Zambia and South Africa. Despite this, data in terms of the prevalence rates in Africa has always been missing [13, 14]. According to the World Health Organization, the prevalence of ASD in many low- and middle-income countries is as yet unknown [8]. Most studies on ASD in Africa have centred on clinical characteristics indicating similarities of children with ASD in Africa and Western world [15-18]. The indication is that most of the children with ASD in Africa have Intellectual Disability (ID), severe speech delay and mostly diagnosed after age eight [19]. This situation is not different from what pertains in Ghana with respect to data on children with ASD. Centers for Disease Control and Prevention assert that there are few published studies providing the necessary information about ASD in Ghana [19]. However, they pointed out that there exist a number of unpublished documents in the phenomenon which are mostly prepared by Non-governmental Organizations (NGOs), though the focus of such documents is on education and treatment of children with ASD. Other studies on ASD in Ghana have sought to address the speech and language therapy for children with ASD, education for children with ASD and classroom environment for children with ASD [20-22].

Knowledge and research of the phenomenon are in the ascendancy in Europe and North America [14]. This, in turn, makes it a little easier for health workers to identify and proffer the needed remedy for children suffering from ASD. However, it remains unclear whether the same applies to Ghana. It is believed that knowledge and awareness about childhood ASD remain at a lower level in Africa [23, 24]. Studies in Africa have showed low knowledge of ASD among healthcare workers. Research on autism from Nigeria reveals a low-level autism knowledge and awareness even among healthcare workers [14, 23]. This is against the backdrop that knowledge of autism among healthcare providers and identifying challenges associated with its management could facilitate bridging the gap and ensuring better outcomes [25]. In Ghana, the primary factor in the identification of children with autism and ID can be attributed in parts, to lack of health professional’s knowledge on the disease [20-22]. One key issue underlying childhood autism has to do with the early diagnosis of the disease. However, the success of early detection and diagnosis to a large extent hinges on the knowledge of health workers on ASD. Several studies on health professionals’ knowledge and understanding on ASD in Sub Saharan Africa, particularly in Nigeria reveal a dearth of knowledge across health professionals on ASD. The African Network for Prevention and Protection against Child Abuse and Neglect observed that there is low to moderate knowledge about ASD among different categories of health care workers [24]. However, their study found a high level of awareness among psychiatrics. In Nigeria for instance, there have been calls for training of healthcare professionals as well as students pursuing health-related programmes in relation to the identification and care of ASD [26, 27]. This assertion is supported by other studies [23, 26, 28] on the subject of low level of knowledge among health professionals on ASD.

The above could be attributed to two main factors. According to Bakare, the low level of knowledge of ASD among health workers exists as primary health care workers in Africa do not routinely undergo training in identification of Neurodevelopmental Disorders (NDD) including ASD [29]. Bakare, therefore, recommended that continues education should be in place to increase knowledge and awareness about ASD among health care workers in order to enhance early recognition and intervention. The second reason is that it is believed instead of empowering majority, if not all health workers, physicians equipped with some level of knowledge on NDD are those with specialised fields (psychiatry, paediatrics and neurology). Even with these specialised fields, the level of knowledge is still low [14]. Reichow, as well as Newton both, listed limited knowledge among health care providers and a dearth of specialist care services as some of the main issues facing autism in Africa [30, 31]. The situation is quite different from other regions. In a study to evaluate the level of knowledge and awareness of ASD among medical students in London revealed that majority of the respondents have adequate knowledge and awareness of childhood autism but were greatly challenge in the area of early detection and diagnosis [32]. Daley reported that healthcare professionals in India believe that the diagnosis of autism is difficult [33]. According to them, 80% of 165 psychiatrists, 95 psychologists and 677 paediatricians affirmed this assertion. Similarly to London, the knowledge about the phenomenon was quite high, particularly among the paediatricians. Heidgerken concluded in their research consisting of psychiatrists, speech and language pathologists, clinical psychologists, and primary health care providers like family physicians, paediatricians and neurologists that these health workers had adequate knowledge of ASD [34]. This research was conducted in the United States by the Centre for Autism Related Disabilities (CARD).

However, in Pakistan, Rahbar reported that the level of knowledge and awareness of ASD among both health workers and medical students was very low [35]. The level of knowledge and awareness of health workers, especially, paediatricians and psychiatrics cannot be overemphasised. This is because there is an important link between knowledge of ASD and the diagnosis of same. According to Rhoades, the level of physician’s knowledge of ASD greatly influences the average age of diagnosis [36].

## 2 METHODS AND MATERIALS

### 2.1 Location

The study was carried out in Kumasi, the capital and the most populous city in the Ashanti Region of Ghana. Kumasi is situated on a 254 square kilometres land size. The central strategic location of the city makes it easily accessible to other parts of the region and nation at large. The city is divided into nine (9) sub-metros – Asokwa, Bantama, Kwadaso, Manhyia, Nhyiaeso, Oforikrom, Suame, Subin and Tafo – for better administrative purposes. There are about 136 health facilities in Kumasi, the majority of which are privately owned and operated. Notable state-owned health facilities within the city include the 1,200 bed capacity Komfo Anokye Teaching Hospital (KATH), which is the second largest hospital in Ghana. The hospital serves as a teaching hospital responsible for the training of medical students at the School of Medical Sciences at the Kwame Nkrumah University of Science and Technology, also located in Kumasi. It is a referral hospital for the northern part of the country (Brong Ahafo, Northern, Upper East and Upper West Regions) as well as parts of the Central, Western, Eastern and Volta regions. Other hospitals include the Kumasi South Hospital (KSH), the Manhyia Hospital (MH), the Suntreso Government Hospital (SGH) and Tafo Government Hospital (TGH).

### 2.2 Participants and Sampling Methods

Participants for the study consisted of paediatrics and psychiatrics nurses drawn from the Paediatrics and Psychiatry Departments at the five main government hospitals (KATH, KSH, MH, SGH and TGH) at the time of the research. At least, each of the nurses had a diploma in nursing, having completed and obtained this honours from one of the 21 public nursing training colleges in the country. In addition, each of them had a practicing nursing certificate from the Nursing and Midwifery Council. At the time of the study, there were 153 paediatric and 98 psychiatric nurses at sampled hospitals. The entire 251 nurses were involved in the study.

### 2.3 Materials

Two main instruments were employed in eliciting data from the paediatric and psychiatric nurses. These were the Socio-demographic and the Knowledge about Childhood Autism among Health Workers (KCAHW) questionnaires.

#### 2.3.1 Socio-Demographic Questionnaire

This questionnaire was used to obtain information on respondents’ gender, age, marital status, working experience, religion and nurses’ previous encounter with children with ASD (Table **1**). 

#### 2.3.2 Knowledge About Childhood Autism among Health Workers (KCAHW) Questionnaire

The KCAHW questionnaire is a self-administered questionnaire that was developed by a team of psychiatrists and clinical psychologists in 2008 at Enugu, Nigeria. It contains a total of nineteen questions. The KCAHW questionnaire has been used in several studies and has been established to have good test-retest reliability, good overall internal consistency (Cronbach’s alpha value of 0.97) and culturally valid [23]. Each of the nineteen items has three options to choose from with only one out of the three being correct. The correct option on each item attracts a score of 1, whereas the other two incorrect options are scored 0 each. The KCAHW questionnaire is divided into the following four domains.

- Domain 1: This domain contains eight items that address the impairments in social interaction usually found in children with childhood autism. A maximum score of 8 and minimum score 0 are possible in this domain;
- Domain 2: This domain contains only one item that addresses impairment in the area of communication and language development, as part of the symptoms seen in children with childhood autism. A maximum score of 1 and a minimum score of 0 are possible in this domain;
- Domain 3: This domain contains four items that address the area of the obsessive and compulsive pattern of behaviour found in children with childhood autism, a pattern of behaviour which had been described as restricted, repetitive and stereotyped. A maximum score of 4 and a minimum score of 0 are possible in this domain; and
- Domain 4: This domain contains six items that address knowledge on what type of disorder childhood autism is, possible co-morbid conditions and period of onset of childhood autism in affected children. A maximum score of 6 and minimum score 0 are possible in this domain.

A maximum total score of 19 and a minimum total score of 0 are possible when the four domain scores are summed up. The mean total score on the KCAHW questionnaire among a particular sample population is a measure of the level of knowledge about childhood autism among that particular population. A total score of 19, which is the maximum score possible on the KCAHW questionnaire, indicates adequate knowledge of symptoms and signs of autism.

### 2.4 Procedure

The two questionnaires were combined into a single document and administered to the respondents at their respective department. After seeking approval from hospital authorities, a meeting was scheduled to introduce the researcher to the nurses where the intention of the study was made known to them. Afterwards, the questionnaire was completed by the nurses and collected immediately. This was to ensure that respondents did not have the opportunity to discuss the responses among themselves as consulting books and other reference materials prior to completing the questionnaire.

### 2.5 Data Analysis

Data collected from the field were edited, coded and entered into the Statistical Product for Service Package (SPSS version 20) software for processing. The mean score on each of the domains was calculated for both the paediatric and psychiatric nurses. In addition, the relationship between nurses’ socio-demographics and knowledge of ASD was assessed using both t-test and One-Way Analysis of Variance (ANOVA). T-test was used to measure the following because they were measured on a dichotomous scale: sex (1 = male, 2 = female); marital status (1 = unmarried, 2 = married) and previous encounter (1 = yes, 2 = no). Since the remaining socio-demographics characteristics (age and working experience) were measured on interval scales, it became eminent to employ ANOVA.

## 3 RESULTS

### 3.1 Socio-Demographic Characteristics of Respondents

A total of 153 and 98 questionnaires were given out to paediatric and psychiatric nurses respectively. Out of all this, 130 and 93 questionnaires from the paediatric and psychiatric nurses were respectively useful for analysis. Overall, the response rate was 88.8%, which is statistically acceptable. There were 84.6% female and 15.4% male paediatric nurses whilst 66.7% female and 33.3% male psychiatric nurses. Exactly half (50%) paediatric nurses were less than 30 years whilst 47.3% psychiatric nurses were less than 30years. 65.4% and 57.0% of paediatric and psychiatric nurses respectively were married. 42.3% of paediatric nurses had worked 5-10 years whilst 43.0% of psychiatric nurses had worked for between 5 and 10years. 58.5% and 61.3% of paediatric and psychiatric nurses respectively have had the previous encounter with children with ASD.

### 3.2 Paediatric and Psychiatric Nurses’ knowledge of ASD

The KCAHW was administered since it is used to assess baseline knowledge about childhood autism among health workers. The total mean score on the KCAHW questionnaire among a particular sample population is a measure of level of knowledge about childhood autism among that particular population. The mean score of respondents (paediatric and psychiatric nurses) on each of the domain as well as the overall mean score on the KCAHW questionnaire are contained in Table (**2**). The mean score for paediatric nurses on Domain 1 which deals with impairments in social interactions was 6.60 ± 0.52 whilst that of psychiatric nurses was 6.71 ± 0.55. On Domain 2 which addresses communication impairments, paediatric nurses had a mean score of 0.92 ± 0.49 with psychiatric nurses scoring 0.94 ± 0.50. With respect to obsessive and repetitive behaviour patterns (Domain 3), the mean score was as follows: paediatric nurses (1.77 ± 0.57) and psychiatric nursed (2.01 ± 1.72). Domain 4 is a measure of the type of disorder childhood autism and possible associated co-morbidity yielded. On this, paediatric nurses had a mean score of 2.08 ± 0.71 whilst psychiatric nursed scored 2.45 ± 1.72. Finally, it can be observed from Table (**2**) that the total mean score for paediatric nurses was 11.37 ± 2.29 whilst psychiatric nurses had 12.11 ± 3.35 as their total mean score. The total mean score, as well as the scores for each of the domains, were higher for psychiatric nurses than that of paediatric nurses.

### 3.3 Socio-Demographic Implications of Paediatric and Psychiatric Nurses’ Knowledge of ASD

The relationship between paediatric and psychiatric nurses’ knowledge on ASD by their socio-demographic variables (gender, age, marital status, working experience and previous encounter with children with ASD) were explored with the aid of the independent sample t-test and One Way Analysis of Variance (ANOVA). Whilst the t-test was conducted on gender, marital status and previous encounter with children with ASD, the ANOVA was conducted on age and working experience. There is no statistically significant difference between both paediatric and psychiatric nurses’ gender and their knowledge of ASD (paediatric nurses: *p*=0.57; psychiatric nurses: *p*=0.49). Similar results were recorded for age (paediatric nurses: *p*=0.80; psychiatric nurses: *p*=0.48), marital status (paediatric nurses: *p*=0.56; psychiatric nurses: *p*=0.47) and working experience (paediatric nurses: *p*=0.47; psychiatric nurses: *p*=0.49). However, there was a significant difference between respondents’ previous encounter with children with ASD and their knowledge of ASD (paediatric nurses: *p*=0.01; psychiatric nurses: *p*=0.01) (Table **3**).

Aside respondents’ socio-demographic characteristics and knowledge of ASD, the study also explored the relationship between respondents’ area of speciality and their knowledge of ASD. The results (as indicated in Table (**4**) returned significant differences for nurses’ area of speciality and knowledge of ASD (F=2.202, p=0.006).

## 4 DISCUSSIONS

Females were more than males with both paediatric and psychiatric nurses. This trend is not surprising since nursing is a female-dominated profession with few males. According to Hsu H, nursing was established through the efforts of Florence Nightingale in the mid-nineteenth century as a women’s profession [37]. Even though more women are found in male-dominated employment, little is seen with respect to men entering traditional female-dominated professions such as nursing [38]. Bureau’s Industry and Occupational Statistics indicate that men represent 9% of registered nurses in the United States, emphasising the fact that nursing is female dominated profession [39]. Two different studies on nursing in Ghana indicate similar trends. In his work, Boafo reported that 80% of his population were female [40]. Similarly, Ofori had 29 females and 21 males in a study conducted at the University of Ghana Nursing School [41]. Though the majority of paediatric and psychiatric nurses had previously encountered children with ASD, 87.7% of paediatric with the previous encounter did not frequently have such encounter. All psychiatric nurses with the previous encounter did not frequently meet children with ASD. The plausible reason accounting for the infrequent encounters can be explained from the fact that the prevalence rate of ASD in Africa and Ghana is very low [12, 28] as compared to prevalence rates outside Africa [9, 42]. Again, Owusu observed that though there are limited resources for the treatment of ASD in Ghana, parents have difficulty accessing these limited resources due to distance, time constraints and lack of information on the availability of these resources [22].

The total mean score on the KCAHW questionnaire was 11.74 ± 2.82 for both paediatric and psychiatric nurses out of a possible total mean score of 19. However, paediatric nurses’ total mean score was 11.37 ± 2.29 whilst that of psychiatric nurses’ was 12.11 ± 3.35. The implication is that paediatric and psychiatric nurses’ knowledge of ASD are minimal. Comparatively, previous studies on health workers knowledge on ASD were higher than the current study. For example, Eseigbe recorded a mean score of 13.5 ± 3.7 among medical doctors in Kaduna, Nigeria [25]. According to Igwe, the total mean score for paediatric and psychiatric nurses on the KCAHW questionnaire was 12.56 ± 3.23 [28]. Again, the total mean score for paediatric and psychiatric nurses was found to be 12.35 ± 4.40 [23]. The foregoing discussions affirm the assertions of Bakare and African Network for the Prevention and Protection against Child Abuse and Neglect that knowledge of ASD in Africa, especially, Nigeria and other sub-Saharan African countries remain lower when compared to those in America, Canada and Europe [23, 24].

A significant difference (F=2.202, p=0.006) was reported on nurses’ area of speciality and their knowledge of ASD. Psychiatric nurses with a total mean score of 12.11 ± 3.35 are perceived to have more knowledge than paediatric nurses with a total means score of 11.37 ± 2.29; confirming the study of Igwe on paediatric and psychiatric nurses’ knowledge on ASD. Since knowledge on ASD leads to early identification [28], it presupposes that psychiatric nurses (with higher knowledge) will probably in a better situation to identifying ASD among children than paediatric nurses (with lower knowledge). Psychiatric nurses had better knowledge than paediatric nurses on all the four domains on the KCAHW questionnaire. With respect to impairments in social interactions, psychiatric nurses scored 6.71 ± 0.55, while paediatric nurses scored 6.60 ± 0.52. Similar trends were observed in communication impairments (Domain 2), obsessive and repetitive behaviour patterns (Domain 3) and type of disorder ASD are possibly associated co-morbidity yielded (Domain 4). Similarly, paediatric nurses scored higher mean score than psychiatric nurses on all four domains in a study in Ebonyi State, Nigeria [28].

Apart from paediatric and psychiatric nurses’ previous encounter with children with ASD (paediatric nurses: *p*=0.01; psychiatric nurses: *p*=0.01), there was no significant difference between the remaining socio-demographic variables (gender, age and working experience) and knowledge of ASD. The implication is that previous encounter with children with ASD influences nurses knowledge about ASD. Specifically, more psychiatric nurses (61.9%) have had the previous encounter with children with ASD than paediatric nurses (58.5%). This confirms the findings of Igwe that there is a statistical difference between the previous encounter with ASD and knowledge of ASD. The plausible reason for this trend is that nurses who have had the previous encounter might have observed the characteristics of ASD. Coupled with theoretical knowledge of ASD, such nurses are in a better position to have adequate knowledge on ASD than those without a previous encounter.

## CONCLUSION

The results of the study indicated a high-knowledge of ASD among paediatric nurses as compared to psychiatric nurses. However, the overall knowledge level among both nursing groups is very low, raising concerns with regards to identification of the disorder among these nurses. Apart from the previous encounter with children with ASD, other demographic factors do not influence knowledge of ASD. Further studies into how paediatric and psychiatric nurses identify ASD should be conducted. Such studies should focus on the analysis of previous background training of nurses and knowledge of ASD.

## Figures and Tables

**Table 1:** Socio-demographic characteristics of respondents.

Socio-demographics Variables	Paediatric Nurses		Psychiatric Nurses	
	Frequency	Percentage	Frequency	Percentage
Gender Male Female Total	20 110 130	15.4 84.6 100.0	31 62 93	33.3 66.7 100.0
Age <30 30-39 >39 Total	65 45 20 130	50.0 34.6 15.4 100.0	44 35 14 93	47.3 37.6 15.1 100.0
Marital Status Single Married Total	45 85 130	34.6 65.4 100.0	40 53 93	43.0 57.0 100.0
Religion Christianity Islam Traditional Total	110 17 3 130	84.6 13.1 2.3 100.0	84 2 - 93	90.3 9.7 - 100.0
Working Experience <5years 5-10years >10years Total	52 55 23 130	40.0 42.3 17.7 100.0	40 40 13 93	43.0 43.0 14.0 100.0
Previous Encounter Yes No Total	76 54 130	58.5 41.5 100.0	57 36 93	61.3 38.7 100.0

**Table 2:** Paediatric and psychiatric nurses’ knowledge of ASD.

Domains	Possible Score	Paediatric	Psychiatric
Domain 1	8	6.60 ± 0.52	6.71 ± 0.55
Domain 2	1	0.92 ± 0.49	0.94 ± 0.50
Domain 3	4	1.77 ± 0.57	2.01 ± 0.58
Domain 4	6	2.08 ± 0.71	2.45 ± 1.72
**Total mean score**	**19**	**11.37 ± 2.29**	**12.11 ± 3.35**

**Table 3:** Knowledge of ASD by respondents’ socio-demographic characteristics.

Respondents’ Profile	Number	Paediatric Nurses	Number	Psychiatric Nurses
Gender Male Female	20 110	2.24 2.26 *p=0.58*	31 62	1.51 1.47 *p=0.49*
Age <30 30-39 >39	65 45 20	2.24 2.26 2.22 *p=0.80*	44 35 14	1.51 1.49 1.41 *p=0.47*
Marital Status Single Married	45 85	2.26 2.28 *p=0.53*	40 53	1.50 1.46 *p=0.47*
Working Experience <5years 5-10years >10years	52 55 23	2.44 2.21 2.03 *p=0.47*	40 40 13	1.49 1.53 1.23 *p=0.49*
Previous Encounter Yes No	76 54	2.33 2.02 p=0.01*	57 36	1.51 1.47 p=0.01*

*Significance level p < .05.

**Table 4:** Knowledge of ASD by respondents’ area of speciality.

**Speciality**	Number	**Total Mean Score**	**p-value/F-Test**
Paediatric nurses	130	11.37 ± 2.29	p=0.006* F=2.202
Psychiatric nurses	93	12.12 ± 3.35	

*Significance level p < .05.
